# Dysregulated Expression of Arterial MicroRNAs and Their Target Gene Networks in Temporal Arteries of Treatment-Naïve Patients with Giant Cell Arteritis

**DOI:** 10.3390/ijms22126520

**Published:** 2021-06-17

**Authors:** Tadeja Kuret, Katja Lakota, Saša Čučnik, Vesna Jurčič, Oliver Distler, Žiga Rotar, Alojzija Hočevar, Snežna Sodin-Šemrl, Mojca Frank-Bertoncelj

**Affiliations:** 1 Institute of Cell Biology, Faculty of Medicine, University of Ljubljana, 1000 Ljubljana, Slovenia; tadeja.kuret@mf.uni-lj.si; 2 Department of Rheumatology, University Medical Centre Ljubljana, 1000 Ljubljana, Slovenia; katja.lakota@guest.arnes.si (K.L.); sasa.cucnik@kclj.si (S.Č.); ziga.rotar@kclj.si (Ž.R.); alojzija.hocevar@gmail.com (A.H.); 3 Faculty of Mathematics, Natural Sciences and Information Technology, University of Primorska, 6000 Koper, Slovenia; 4 Faculty of Pharmacy, University of Ljubljana, 1000 Ljubljana, Slovenia; 5 Institute of Pathology, Faculty of Medicine, University of Ljubljana, 1000 Ljubljana, Slovenia; vesna.jurcic@mf.uni-lj.si; 6 Center of Experimental Rheumatology, Department of Rheumatology, University Hospital Zurich, University of Zurich, 8952 Schlieren, Switzerland; oliver.distler@usz.ch; 7 Faculty of Medicine, University of Ljubljana, 1000 Ljubljana, Slovenia

**Keywords:** giant cell arteritis, microRNA, microRNA-target genes, arterial remodelling, toll-like receptor signaling, arterial ultrasound, vision disturbances

## Abstract

In this study, we explored expression of microRNA (miR), miR-target genes and matrix remodelling molecules in temporal artery biopsies (TABs) from treatment-naïve patients with giant cell arteritis (GCA, *n* = 41) and integrated these analyses with clinical, laboratory, ultrasound and histological manifestations of GCA. NonGCA patients (*n* = 4) served as controls. GCA TABs exhibited deregulated expression of several miRs (miR-21-5p, -145-5p, -146a-5p, -146b-5p, -155-5p, 424-3p, -424-5p, -503-5p), putative miR-target genes (*YAP1*, *PELI1*, *FGF2*, *VEGFA*, *KLF4*) and matrix remodelling factors (*MMP2*, *MMP9*, *TIMP1*, *TIPM2*) with key roles in Toll-like receptor signaling, mechanotransduction and extracellular matrix biology. MiR-424-3p, -503-5p, *KLF4*, *PELI1* and *YAP1* were identified as new deregulated molecular factors in GCA TABs. Quantities of miR-146a-5p, *YAP1*, *PELI1*, *FGF2*, *TIMP2* and *MMP9* were particularly high in histologically positive GCA TABs with occluded temporal artery lumen. MiR-424-5p expression in TABs and the presence of facial or carotid arteritis on ultrasound were associated with vision disturbances in GCA patients. Correlative analysis of miR-mRNA quantities demonstrated a highly interrelated expression network of deregulated miRs and mRNAs in temporal arteries and identified *KLF4* as a candidate target gene of deregulated miR-21-5p, -146a-5p and -155-5p network in GCA TABs. Meanwhile, arterial miR and mRNA expression did not correlate with constitutive symptoms and signs of GCA, elevated markers of systemic inflammation nor sonographic characteristics of GCA. Our study provides new insights into GCA pathophysiology and uncovers new candidate biomarkers of vision impairment in GCA.

## 1. Introduction

Giant cell arteritis (GCA) is a systemic vasculitis that occurs in individuals older than 50 years and affects large- and medium-sized arteries, especially the extracranial branches of the carotid artery and the aorta [1]. GCA is characterized by ischaemic manifestations such as headache, jaw claudication, visual disturbances, and limb ischemia, and is accompanied by symptoms and signs of systemic inflammation that include fatigue, fever, weight loss, myalgia and increased inflammatory markers (erythrocyte sedimentation rate (ESR), C-reactive protein (CRP) and serum amyloid A (SAA)) [2,3,4].

The temporal artery biopsy (TAB) showing a transmural inflammatory infiltrate enables diagnosis of GCA [5]. Nonetheless, a histologically negative TAB does not exclude GCA. The vascular involvement in GCA can be segmental, and the inflammatory infiltrates are limited to adventitia in individual patients [6,7]. Since TAB is invasive, it is being increasingly replaced by arterial imaging using different imaging modalities, particularly colour Doppler ultrasonography of the arteries [8]. The use of ultrasound, however, requires skilled sonographers and is not yet widespread in routine clinical practice [9].

Research, performed on TABs has contributed significantly to current understanding of inflammatory and remodelling events in GCA arteries that ultimately lead to ischaemic disease manifestations [10]. The current pathogenesis concept of GCA suggests initial activation of *vasa vasorum* and dendritic cells in the adventitia of the affected arteries, followed by infiltration, activation and differentiation of CD4^+^ T-cells into IFNγ-secreting Th1 cells and IL-17-secreting Th17 cells. Subsequently, monocytes are recruited to the arterial media, differentiating into macrophages and forming multinucleated giant cells [11]. Inflammatory infiltrates produce copious amounts of inflammatory cytokines, chemokines, growth factors and proteolytic enzymes which drive local inflammatory response [12]. Arterial stromal cells, including vascular smooth muscle cells (VSMCs) and the endothelial cells of the *vasa vasorum* (the outside-in concept of inflammation) [13], play a significant role in vascular inflammation and remodelling in GCA [14]. VSMCs undergo a phenotypic switch into highly proliferative synthetic cells [15], which are, together with macrophages, a rich source of reactive oxygen species, matrix metalloproteinases (MMPs, particularly MMP2 and MMP9) and growth factors (vascular endothelial growth factor (VEGF), platelet-derived growth factor). These molecules contribute to destruction of arterial wall structures, support progressive adventitial neovascularization and drive rapidly evolving intimal hyperplasia and arterial occlusion [16,17,18]. As the final effectors of the pathogenic cascade, VSMCs represent a candidate therapeutic target in GCA [15].

MicroRNAs (miRs) are short non-coding regulatory RNAs that influence gene expression post-transcriptionally by controlling messenger RNA (mRNA) translation and/or inducing mRNA degradation [19]. A single miR can regulate the expression of different target mRNAs and individual mRNAs can be targeted by many miRs [20]. While miRs commonly negatively regulate their target genes, some miRs also act as positive regulators of their target gene expression [21]. Additionally, miRs exert many indirect effects on gene regulation, for example by influencing the expression of other miRs and transcriptional factors [22]. Deregulated expression of miRs and their target genes significantly contributes to the pathogenesis of cardiovascular diseases, including different autoimmune vasculitides (e.g., Kawasaki disease, Behcet’s disease, GCA) [23,24].

To date, two studies have investigated miR expression in TABs of GCA patients [25,26]. Croci et al. [25] measured the expression of 1209 miRs in histologically positive TABs of GCA patients compared to TAB negative nonGCA controls. This study identified six significantly altered miRs in TABs of GCA patients including miR-21, -146a, -146b-5p, -150, -155, and -299-5p, suggesting a deregulation of miRs in GCA arterial lesions. GCA patients enrolled in this study received the steroid treatment prior to TAB sampling [25]. Meanwhile, Bolha et al. [26] studied the expression of 752 miRs in TABs from treatment-naïve GCA patients. This study demonstrated enhanced expression of miRs promoting the synthetic VSMC phenotype (miR-21-5p, -146a-5p, -146b-5p and -424-5p) and under-expression of miRs promoting the contractile VSMC phenotype (miR-23b-3p, -125a-5p, -143-3p, -143-5p, -145-3p, -145-5p, -195-5p, -365a-3p) in GCA. The authors also identified in silico the candidate target genes of deregulated miRs, mainly involved in arterial wall remodelling and immune system regulation [26].

Here, we explored the expression networks of miRs, miR-target genes and matrix remodelling genes in TABs from treatment-naïve GCA patients and linked the expression of these molecules to clinical, ultrasound and histological features of GCA. Our study provides new insights into GCA pathogenesis and identifies potential new biomarkers of vision impairment in GCA.

## 2. Results

### 2.1. Patient Characteristics and Establishment of GCA Diagnosis

**Figure 1:**
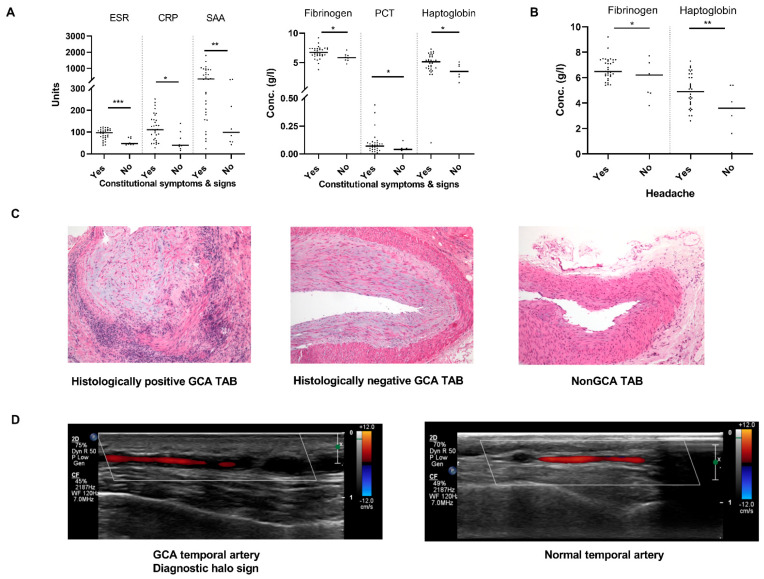
Histological, clinical and laboratory characteristics of GCA patients and nonGCA control subjects. (**A**,**B**) Differences in systemic inflammatory markers in TAB positive GCA patients (**A**) with and without constitutional symptoms and signs and (**B**) with and without headache. Units used to measure ESR, CRP and SAA in (**A**) were mm/h, g/L, mg/L, respectively. (**C**) Representative HE stainings of histologically positive and negative GCA TABs and normal TABs from nonGCA subjects. Inflammatory cell infiltrates with granulomas are present in histologically positive GCA TABs, but not histologically negative GCA TABs and nonGCA TABs. Histologically positive GCA TABs demonstrate extensive intimal hyperplasia, there is a marked intimal hyperplasia in histologically negative GCA TABs, while the intima is rather thin in non-GCA TABs. (**D**) Representative ultrasound imaging scans of temporal arteries in GCA patients (inflamed temporal artery with a diagnostic halo sign) and control nonGCA subjects (normal temporal artery without a halo sign) * *p* < 0.05; ** *p* < 0.01; *** *p* < 0.001. Legend: ESR, erythrocyte sedimentation rate; CRP, C-reactive protein; SAA, serum amyloid A; GCA, giant cell arteritis; HE, hematoxylin and eosin; PCT, procalcitonin; TAB, temporal artery biopsy.

Our study enrolled forty-one treatment-naïve GCA patients (26/41 females, median age 77 years, Q_25_–Q_75_ 71–79 years) and four nonGCA patients (2/4 females, median age 80 years, Q_25_–Q_75_ 71–83 years). Detailed clinical, laboratory and histological characteristics of GCA patients are shown in Table 1. Briefly, GCA patients presented with variable constitutional signs and symptoms (weight loss, fatigue, fever, sweating, myalgias), ischaemic complications (new onset headache, jaw claudication, vision disturbances, vision loss) and increased systemic inflammatory markers (Table 1). Systemic inflammatory markers were particularly high in GCA patients with constitutional manifestations (Figure 1A) and headache (Figure 1B). The four nonGCA patients, who presented with variable clinical signs and symptoms that raised suspicion of GCA, were finally diagnosed with other illnesses: ANCA vasculitis, lymphoproliferative disease, pan-arteritis nodosa and prostate carcinoma.

Thirty-five (85.4%) GCA patients had positive TAB histology showing a diagnostic transmural mononuclear inflammatory cell infiltration, while TAB was negative in 14.6% (*n* = 6) of GCA patients (Table 1, Figure 1C). Systemic inflammatory markers were comparably high in GCA patients with positive and negative TABs (Supplementary Figure S1A). Temporal artery lumen occlusion was present in 51% of histologically positive GCA TABs and none of histologically negative GCA TABs (*p* = 0.020, Table 1, Figure 1C). The presence of temporal artery lumen occlusion in positive GCA TABs was not associated with constitutional manifestations, ischaemic complications (data not shown) nor systemic inflammatory markers (Supplementary Figure S1B). Colour Doppler ultrasonography of temporal arteries detected a diagnostic halo sign (Figure 1D) in thirty-three GCA patients with positive TAB and in all GCA patients with histologically negative TAB, pointing to a GCA diagnosis in 95% of patients with suspicion of GCA (Table 1). Temporal artery thickness, as measured by ultrasound, did not differ between GCA patients with histologically positive and negative TABs (Supplementary Figure S1C) nor between histologically positive GCA patients with and without lumen occlusion on TAB (Supplementary Figure S1D).

### 2.2. Sonographic Pattern of GCA Affected Arteries Associates with Ischaemic Complications

We performed extended ultrasound imaging of cranial (temporal, vertebral, occipital, facial) and large noncranial (carotid, axillary, subclavian) arteries that are characteristically affected by GCA (Table 1). Twenty-eight GCA patients (68.3%) showed extended cranial artery involvement (≥2 affected cranial arteries) and eight GCA patients (19.5%) had ultrasound evidence of accompanying large vessel vasculitis (Table 1). Extended cranial and axillar arteritis associated with a higher frequency of jaw claudication and headache, respectively, while visual disturbances occurred more often in GCA patients with facial and carotid artery involvement (Table 2). Sonographic patterns of arterial involvement did not associate with constitutional symptoms, increase in systemic inflammatory markers nor with histologically measured lumen occlusion on TABs. Lack of the association between sonographic measures of arterial thickness and histological measures of lumen occlusion might reflect the differences in the arterial substrate assessed by these two modalities. Additionally, histology is done on excised arteries, which can deform to a certain extent during excision and experimental procedures.

### 2.3. MiR-424-3p and miR-503-5p Are Novel Deregulated miRs in GCA TABs

Deregulated miR expression in diseased tissues may provide important insights into pathogenic pathways and altered moleular networks. To explore whether and how arterial pathology in GCA alters miR expression, we compared miR expression in positive GCA TABs, negative GCA TABs and TABs from nonGCA individuals. Our analysis included miRs that play roles in immune response and inflammation, VSMC functions and angiogenesis [27,28,29], covering a range of deregulated biological pathways in GCA. We measured miRs that were shown as deregulated in GCA TABs in previous studies [25,26] (miR-21-5p, -23b-5p, -125a-5p, -143-5p, 145-5p, 146a-5p, -146b-5p, -155-5p, -195-5p and -424-5p) and added new miR candidates, including miR-17-3p, -27b-5p, 125b-5p, -126-3p, -130a-5p, -424-3p and -503-5p. These new miR candidates were selected based on the literature search for known miR roles in biological processes, relevant to the pathogenesis of GCA [18,27,28,29,30].

**Figure 2:**
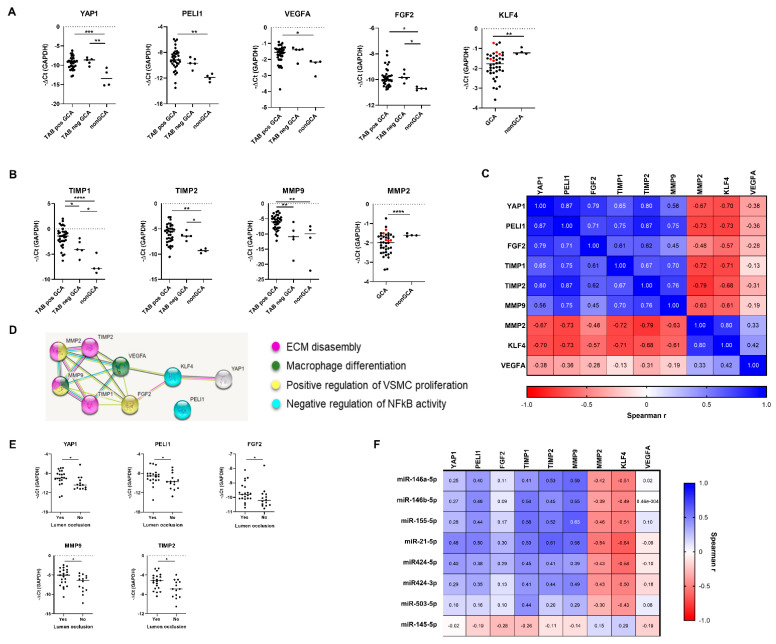
Deregulated miR expression in TABs from GCA patients. (**A**) Differentially expressed miRs in GCA patients with histologically positive TABs (*n* = 35), GCA patients with histologically negative TABs (*n* = 6) and TABs from nonGCA subjects (*n* = 4). miR expression was calculated as negative delta Ct (−ΔCt) of individual miRs normalized to RNU48 expression. Line represents the median miR expression in each patient group. (**B**) Correlation between normalized expression of miR-424-3p, -424-5p and -503-5p in TABs from GCA and nonGCA patients. (**C**) Matrix of Spearman’s rank correlation coefficients between normalized miR expression across GCA (*n* = 41) and nonGCA (*n* = 4) TABs. Plotted are miRs that were identified as deregulated in GCA TABs. *p* values of the correlations are provided in the Supplementary Table S1. (**D**) Normalized expression of miR-146a-5p in histologically positive GCA TABs with or without temporal artery lumen occlusion. (**E**). Matrix of Spearman’s rank correlation coefficients between normalized miR expression and sonographic measurements of the temporal arterial thickness in 41 GCA patients. Plotted are miRs that were identified as deregulated in GCA TABs. (**F**) Matrix of Spearman’s rank correlation coefficients between normalized miR expression and systemic inflammation markers in 35 GCA patients with histologically positive TABs. Plotted are miRs that were identified as deregulated in GCA TABs. *p*-value of <0.05 was considered as statistically significant. * *p* < 0.05; ** *p* < 0.01; *** *p* < 0.001; **** *p <* 0.0001. Legend: CRP, C-reactive protein; ESR, erythrocyte sedimentation rate; GCA, giant cell arteritis; HB, heamoglobin; miR, microRNA; PCT, procalcitonin; SAA, serum amyloid A; TAB, temporal artery biopsy.

We confirmed the deregulated expression of miR-21-5p, -145-5p, -146a-5p, -146b-5p, -155-5p and -424-5p in positive GCA TABs (Figure 2A) compared to histologically negative GCA TABs and/or nonGCA TABs as also shown in previous studies [25,26]. The functional roles of deregulated miRs are described in Supplementary Table S1. The deregulation of other previously reported miRs including miR-23b-5p, -125a-5p, -143-5p and -195-5p was not confirmed in our study (Supplementary Figure S2A,B).

Among the measured new candidate miRs, we identified miR-424-3p as significantly upregulated miR in histologically positive TABs from GCA patients compared to histologically negative TABs and TABs from nonGCA individuals (Figure 2A), whereas miR-503-5p differed significantly between histologically positive and negative GCA TABs (Figure 2A). MiRs-503 and -424 are encoded as a miR cluster in the genomic region of the long noncoding RNA H19X gene also known as MIR503HG [31]. Our analysis showed a strong correlation between miR-424-3p/-5p and miR-503-5p expression in GCA and nonGCA TABs (Figure 2B). No correlation was observed between these miRs and H19X expression (Supplementary Figure S2C). The levels of miRs-17-3p, -27b-5p, -125b-5p, -126-3p and -130a-5p (Supplementary Figure S2A,B) and H19X (Supplementary Figure S2D) did not differ between the patient groups.

### 2.4. Deregulated miRs Exhibit a Highly Interrelated Expression Signature in GCA TABs

The upregulated miRs showed a strongly interrelated expression pattern across GCA and nonGCA TABs (Figure 2C, Supplementary Table S2), particularly miR-21-5p/146a-5p/-155-5p and miR-21-5p/-424-3p/-424-5p. The deregulation of these miRs was observed solely in patients with positive GCA TABs (Figure 2A). Furthermore, miR-146a-5p expression was significantly increased in positive GCA TABs exhibiting temporal artery lumen occlusion (Figure 2D) compared to non-occluded positive GCA TABs. Deregulated miR expression did not correlate with sonographic measurements of the temporal artery thickness (Figure 2E) in GCA patients. No correlation was found between the deregulated miR expression and eleveated systemic inflammatory markers (Figure 2F) in TAB positive GCA. Overall, these data suggested that the local molecular or cellular factors in the arterial wall significantly contributed to altered miR expression in GCA TABs.

### 2.5. Analysis of miR-Target Genes Identifies PELI1, YAP1 and KLF4 as New Deregulated Genes in GCA TABs

Next, we explored whether target genes of derugulated miRs are differentially expressed in TABs across patient groups. Target genes of deregulated miR-21-5p, -145-5p, 146a-5p, -146b-5p, -155-5p, 424-3p/5p and -503-5p were selected based on the miRror database [32] of experimentally validated miR-target gene pairs and literature search (Supplementary Table S3). We focused on genes with potential relevance to GCA pathobiology, including genes involved in VSMC function, angiogenesis and tissue remodelling. Additionally, we analysed the expression of key tissue remodelling factors, specifically MMPs 2, 9, 14 and tissue inhibitors of MMPs (TIMP 1, 2), previously shown to be altered in GCA arteries [33,34,35]. This analysis enabled us to get insights into the arterial wall remodelling proces in our GCA patient cohort and associate the findings with the expression changes of miRs and their target genes in GCA TABs. Cellular origins and functions of deregulated miR-target genes and matrix remodelling genes are described in Supplementary Table S1.

**Figure 3:**
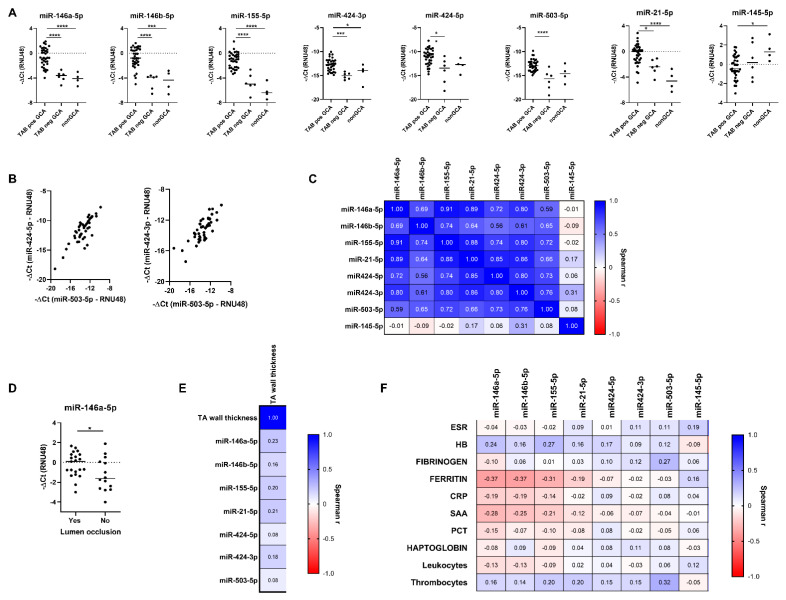
Deregulated expression of miR-target genes and matrixremodelling genes in TABs from GCA patients. (**A**,**B**) Differentially expressed (**A**) miR-target genes (see also Supplementary Table S3) and (**B**) matrix-remodelling genes in histologically positive GCA TABs (*n* = 35), histologically negative GCA TABs (*n* = 6) and nonGCA TABs (*n* = 4). Gene expression was calculated as negative delta Ct (−ΔCt) of individual mRNAs normalized to GAPDH expression. Line represents the median mRNA expression in each patient group. Values for GCA patients with negative TABs are marked with red. (**C**) Matrix of Spearman’s rank correlation coefficients between normalized expression of miR-target genes and matrix-remodelling genes across GCA (*n* = 40) and nonGCA (*n* = 4) TABs. Plotted are mRNAs that were identified as deregulated in GCA TABs. *p* values of the correlations are provided in the Supplementary Table S2. (**D**) STRING enrichment analysis of functional and physical protein-protein networks of deregulated mRNAs in GCA TABs using the medium confidence 0.400 for the minimimum required interaction score. Colour symbols indicate GCA-relevant GO biological processes that were among the most significantly enriched (FDR < 0.05). (**E**) Normalized expression of miR-target genes and matrix-remodelling genes in histologically positive GCA TABs with or without temporal artery lumen occlusion. (**F**) Highly interrelated expression of deregulated miRs, miR-target genes and matrix-remodelling genes in temporal arteries from GCA (*n* = 40) subjects. Matrix of Spearman’s rank correlation coefficients between normalized expression of miRs, miR-target genes and matrix-remodelling genes across GCA (*n* = 40) and nonGCA (*n* = 4) TABs. Plotted are miRs and mRNAs that were identified as deregulated in GCA TABs. Correlative analyses are based on Sperman’s correlation coefficients with *p* < 0.05 considered as statistically significant. *p* values of the correlations are provided in the Supplementary Table S1. * *p* < 0.05; *** *p* < 0.001; **** *p* < 0.0001. Legend: YAP1, Yes-associated protein 1; PELI1, Pellino E3 ubiquitin protein ligase 1; KLF4, Kruppel-like factor 4; VEGFA, vascular endothelial growth factor A; FGF2, fibroblast growth factor 2; TIMP, tissue inhibitor of matrix metalloproteinase; MMP, matrix metalloproteinase; CRP, C-reactive protein; ESR, erythrocyte sedimentation rate; GCA, giant cell arteritis; HB, heamoglobin; mRNA, messenger RNA; miR, microRNA; PCT, procalcitonin; SAA, serum amyloid A; TA, temporal artery; TAB, temporal artery biopsy.

We showed that the expression of several genes targeted by the deregulated miRs was altered in GCA TABs compared to nonGCA TABs. Specifically, *Yes-asssociated protein 1 (YAP1)*, *Pellino E3 ubiquitin protein ligase 1 (PELI1)*, *vascular endothelial growth factor A (VEGFA)* and *fibroblast growth factor 2 (FGF2)* mRNAs were significantly upregulated in GCA patients with positive TAB compared to nonGCA controls, while the expression of *Kruppel-like factor 4 (KLF4)* mRNA was downregulated in GCA patients compared to nonGCA controls (Figure 3A). *SMAD7*, *SMURF2*, *MYOCD* and *PDCD4* mRNA levels did not differ significantly between the patient groups (Supplementary Figure S3A,B).

The analysis of MMPs and TIMPs showed altered expression of *TIMP1*, *TIMP2* and *MMP9* in positive GCA TABs versus control nonGCA TABs while *MMP2* mRNA expression differed between GCA TABs versus nonGCA TABs (Figure 3B), pointing to an active tissue remodelling process in GCA temporal arteries. *MMP14* mRNA did not differ significantly across patient groups (Supplementary Figure S3A,B).

Notably, the expresion of *YAP1*, *FGF2*, *TIMP1* and *TIMP2* was increased also in negative GCA TABs compared to control nonGCA TABs (Figure 3A,B), inferring that specific transcriptional changes could occur also in GCA arterial walls devoid of transmural immune cell infiltration.

### 2.6. Deregulated miR-Target Genes and Matrix Remodelling Genes Constitute an Interrelated Gene Network in GCA Arteries

Correlating the expression of deregulated mRNAs uncovered a strongly interrelated mRNA signature across GCA and nonGCA TABs comprising of *YAP1*, *PELI1*, *FGF2*, *MMP9*, *TIMP1* and *TIMP2* mRNAs (Figure 3C, Supplementary Table S2). The interacting functional network of these molecules was shown also by the STRING protein-protein network analysis (Figure 3D).

Together, these findings inferred that a common inciting stimulus, an expanded cellular population or a shared biological pathway related to arterial wall remodelling could underlie the observed gene expression changes in GCA TABs. In line with this, the expression of most interrelated genes was significantly higher in positive GCA TABs with temporal artery lumen occlusion compared to non-occluded positive GCA TABs (Figure 3E). No correlations were observed between deregulated mRNA expression and arterial wall thickness in GCA patients as measured by the ultrasound (Supplementary Figure S3C) nor systemic inflammatory markers (Supplementary Figure S3D). These results aligned with the lacking association between the temporal arterial wall thickness and luminal occlusion in GCA positive TABs (Supplementary Figure S1D).

### 2.7. Correlative Analysis of miR-mRNA Expression Identifies KLF4 as Candidate Target Gene of Deregulated miR Network in GCA TABs

Given the interrelated expression of deregulated miRs (Figure 2C) and mRNAs (Figure 3C) in temporal arteries, we hypothesized that correlative analysis of miR and mRNA expression could capture miR-mediated gene regulation but also reveal miR-mRNA networks concertedly driving the arterial pathology in GCA.

We demonstrated an inverse correlation between *KLF4* mRNA expression and several *KLF4* targeting miRs, including miR-21-5p, -146a-5p and -155-5p (Figure 3F, Supplementary Table S3). Whereas these miRs were upregulated in GCA TABs (Figure 2A), *KLF4* was downregulated (Figure 3A), suggesting that miRs -21-5p, -146a-5p and -155-5p might repress arterial *KLF4* expression. Certain miRs negatively regulate their target genes [21,36], but positive regulatory effects have been described for some miR-target gene pairs [37], including miR-21-driven *PELI1* expression [38]. *YAP1* and *PELI1* mRNA expression correlated positively with their targeting miRs-21-5p/-424-5p and miRs-21-5p/-155-5p (Figure 3F). No correlations were observed between other deregulated miRs and their target genes (Figure 3F).

The expression of deregulated genes (*KLF4*, *PELI1*, *MMP2*, *MMP9*, *TIMP1*, *TIMP2*) often correlated with non-targeting miRs (Figure 3F) across GCA and nonGCA TABs. For example, matrix remodelling mRNAs *MMP9*, *TIMP1* and *TIPM2* correlated with miR-21-5p, -146a-5p, -146b-5p and -155-5p, while *KLF4* mRNA expression was negatively associated with non-targeting miR-146b-5p, -424-3p and -424-5p. These observations suggested that alteration in these miRs and mRNAs may stem from common disease pathway or cellular origins in GCA TABs or capture the secondary miR effects on gene expression.

### 2.8. Temporal Artery Expression of miR-424-5p Is Higher in GCA Patients with Visual Impairment

Our analyses identified an interlinked miR and mRNA expression signatures in GCA TABs (Figure 2C and Figure 3C,F) and showed that many of these molecules were particularly perturbed in GCA patients with temporal artery lumen occlusion (Figure 2D and Figure 3E). In contrast, no associations were found between the deregulated miRs and mRNAs and elevated markers of systemic inflammation (Figure 2F, Supplementary Figure S3D). Based on these findings, we hypothesized that the deregulated arterial miR and mRNA expression might correlate with ischaemic but not constitutional GCA manifestations.

**Figure 4:**
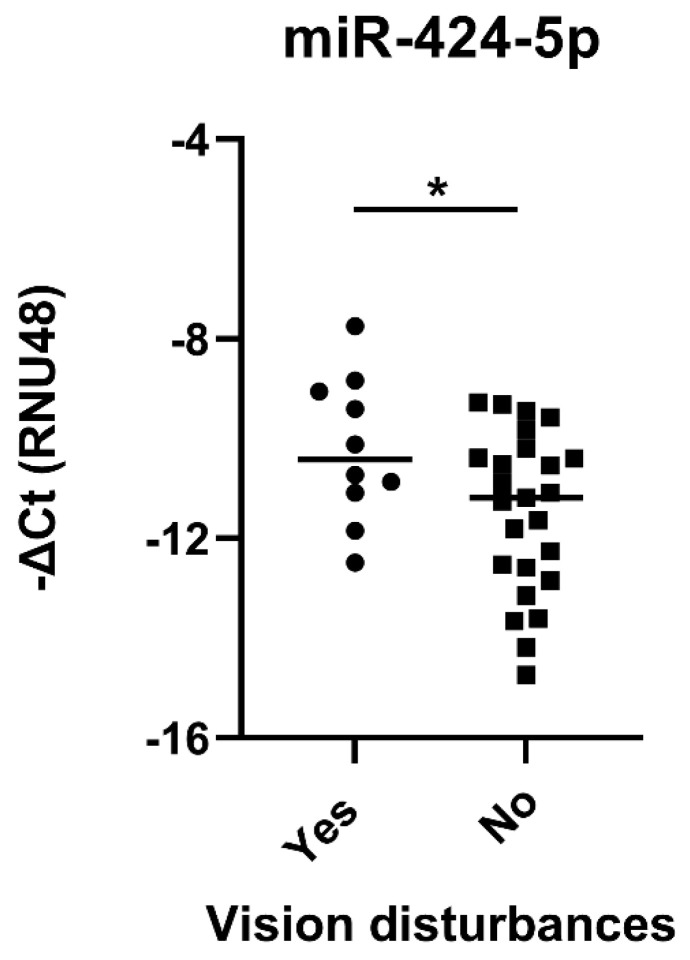
Increased expression of miR-424-5p in histologically positive TABs from GCA patients with vision disturbances. Normalized expression of miR-424-5p in 35 histologically positive TABs from GCA patients with or without vision disturbances. miR expression was calculated as negative delta Ct (−ΔCt) of individual miRs normalized to the RNU48 expression. Line represents the median miR expression in each patient group. * *p* < 0.05. Legend: GCA, giant cell arteritis.

To test this hypothesis we compared miR and mRNA expression in GCA patients with and without ischaemic or constitutional manifestations. GCA patients with negative TAB histology were excluded from this analysis given the segmential lesion nature of GCA and the confirmed affliction of their temporal arteries with ultrasound imaging (Table 1). In line with our hypothesis, the expression of deregulated miRs and mRNAs did not differ with the occurrence of constitutional signs and symptoms of GCA nor the sonographic extent and patterns of arterial involvement. However, the levels of miR-424-5p were significantly increased in TABs from patients with visual disturbances (Figure 4). Other miRs and mRNAs did not correlate with the ischaemic GCA manifestations.

## 3. Discussion

Disturbed expression of miR networks contributes to gene deregulation and pathogenesis of human disease. In the current study, we integrated clinical, laboratory, histological and ultrasound imaging parameters with miR and gene expression analysis of diseased temporal arteries in treatment-naïve GCA patients. The effects of glucocorticoids on gene and miR expression have been shown in a variety of human immune and stromal cell types, many relevant to arterial wall pathology in GCA [39,40,41]. A unique access to glucocorticoid-naïve TABs in our study is based on our health-care model where patients with suspicion of GCA are urgently admitted to rheumatology clinic, where they undergo the diagnostic procedure, including TAB, and initiate therapy on the day of admission. To diagnose GCA, we used TAB (a gold standard) combined with arterial ultrasound imaging and could diagnose GCA in all enrolled patients with GCA. The observed diagnostic discrepancy between the temporal artery ultrasound and TAB histology could arise from the differential inspected length of the affected artery, particularly given the segmental nature of arterial lesions in GCA [6,7].

Combining clinical, imaging and molecular disease characteristic can significantly advance stratification of patients in prognostic and therapeutic groups. In GCA, irreversible vision damage remains the most feared acute complication and identifying GCA patients at risk for vision damage represents a major clinical need [42,43]. Several clinical, laboratory, histological and ultrasound imaging parameters have been associated with increased risk of vision disturbances in GCA. These factors include advanced age, jaw claudication, the presence of giant cells in TABs and increased stroke-risk stratification CHADS_2_ score [44]. Our findings demonstrated that vision impairment was more frequent in GCA patients with sonographic signs of facial or carotid arteritis and increased temporal artery miR-424-5p expression. Prospective studies on larger patient cohorts should validate these findings, evaluating the potential roles of arterial ultrasound imaging and miR-424-5p expression as predictive biomarkers for visual complications in GCA.

In the current study, we undertook a stepwise approach in analyzing perturbed miR-target gene regulation in temporal arteries of patients with GCA. In the first step, the deregulated miRs were identified, subsequently guiding the target gene selection. While facilitating discovery of candidate miR-target gene interactions, this approach proved instrumental in uncovering interrelated miR and mRNA arterial networks and identifying new deregulated molecules in GCA TABs.

Several deregulated miRs, their gene targets and matrix remodelling genes constituted highly interrelated arterial gene expression networks and correlated with histological signs of arterial wall remodelling (lumen occlusion). These findings inferred considerable alterations of the local arterial microenvironment where inflammatory cells and VSMCs could play a potentially pivotal role [45,46,47,48,49]. Single cell RNA-sequencing studies and cell type deconvolution in the bulk transcriptomic data from GCA TABs would be needed to confidently determine the cell type-specific contributions to the identified gene expression changes in GCA TABs in our study.

The observed elevated and highly interrelated expression of miR-21-5p, -146a-5p and -155-5p but also miR-21-5p, -424-3p and -424-5p in GCA TABs might be linked to the enhanced Toll-like receptor (TLR) signaling in GCA TABs [50], accompanied by an expansion of pro-synthetic pathogenic VSMCs and loss of contractile VSMCs in diseased arterial walls [26]. MiR-21, -146a and -155 are aberrantly increased in many autoimmune and inflammatory diseases including rheumatoid arthritis [51,52] and psoriasis [53]. These miRs are commonly upregulated by TLR activation and control TLR signaling via positive and negative feedback loops. Whereas miR-155 accelerates the pro-inflammatory TLR signaling, miR-21 and -146a serve as TLR signaling inhibitors [54]. MiR-146a represses the expression of the downstream components (TRAF6, IRAK1) of the TLR signaling cascade [55] and miR-21 increases IL-10 by inhibiting programmed cell death 4 (PDCD4) [56]. Additionally, miR-21-5p, -146a-5p and -424-5p contribute to the phenotypic switching of VSMC towards the pathogenic synthetic cell phenotype, associated with intimal hyperplasia and vascular occlusion in GCA. Deregulation of these miRs has been recently linked to the increased arterial wall thickness and enhanced ratio between intima and media thickness in GCA TABs [26] and we have shown an elevated expression of miR-146a-5p in occluded histologically positive GCA TABs.

Highly correlated perturbations in *PELI1-YAP1-TIMP2* mRNA expression further supported the alterations in TLR signaling and matrix remodelling pathways in GCA arteries. Namely, the expression of *PELI1* is enhanced in response to TLR ligation and PELI1 exhibits core regulatory roles in fine-tuning the TLR responses [57,58]. In turn, YAP1 plays critical roles in cardiovascular diseases associated with extensive vessel wall remodelling, including atherosclerosis [59], pulmonary hypertension [60,61] and coronary artery restenosis following angioplasty [46] by accelerating migration of VSMC, increasing proliferation of VSMCs, adventitial fibroblasts and endothelial cells and driving ECM deposition and remodelling [62,63]. Alongside *PELI1* and *YAP1*, *KLF4*, miR-424-3p and -503-5p were uncovered as new deregulated mRNAs and miRs in GCA TABs. While this data inferred a general perturbation of the miR-424/503 cluster in GCA TABs, the expression of their host gene *H19X* remained unchanged, suggesting a decoupling of miR-424, miR-503 and *H19X* expression in GCA TABs. MiR-503, miR-424 and *H19X* can be co-expressed, but can perform distinct functions as recently shown in pro-fibrotic skin fibroblasts [64].

Our gene expression analyses showed that several genes, including *YAP1*, *FGF2*, *TIMP1* and *TIMP2* mRNAs were deregulated in histologically negative GCA TABs compared to nonGCA TABs. Given the lack of transmural inflammatory cell infiltration in negative GCA TABs, these changes might mirror deregulation of arterial stromal cells, caused by cell intrinsic mechanisms or extrinsic molecules, diffusing from nearby immune-infiltrated GCA lesions or entering the arterial wall from systemic circulation. Indeed, negative GCA TABs, albeit devoid of transmural immune cell infiltrates, can exhibit early histological signs of arterial wall changes, reflected in an increased arterial wall thickness and enhanced ratio of the intima/media thickness [26].

MiR-driven target gene regulation might be masked in whole tissue analyses given the heterogeneity of cell types, varying cell type frequencies and unequal contribution of these cell types to the total isolated tissue RNA mass. Despite these challenges, we identified *PELI1* as a candidate gene target of deregulated miR-21 [38], and *KLF4* as a potential direct gene target of a deregulated miR-21-5p, -146a-5p and -155-5p network in temporal arteries of patients with GCA. KLF4 is one of the principal mechanosensitive factors in vascular endothelial cells with widespread atheroprotective and vascular homeostatic effects [49,65,66]. The exposure of endothelial cells to laminar flow could upregulate KLF4, while downregulating YAP1 [67]. Furthermore, the mutually inhibitory YAP1-KLF4 interactions were shown to be critical for maintaining the homeostasis of skin [68]. Our results point to upregulated *YAP1* and downregulated *KLF4* mRNAs in GCA TABs, however whether these factors mechanistically interact in diseased arteries to drive GCA pathology needs to be further elucidated. Furthermore, *YAP1* expression corelated positively with miR-21 levels and miR-21-driven RUNX1 repression led to elevated *YAP* expression in myeloid-derived suppressor cells [69].

Our study was undertaken in a single clinical center in Slovenia, which provides a unique access to treatment-naïve TABs, but serves only a population of 320,000 individuals older than 50 years. Therefore, we could enroll only a small number of TABs from nonGCA controls, which represents a limitation of our study. TAB is an invasive procedure and can be performed only in patients with a high suspicion of GCA diagnosis due to ethical reasons. Additionally, over the recent years, we have reached a high diagnostic accuracy in diagnosing GCA by ultrasound, which has further decreased the frequency of TAB in patients with suspected GCA having a negative ultrasound examination. Enrolling a larger number of nonGCA subjects could lead to discovery of additional deregulated miRs and mRNAs in GCA TABs that exhibit a smaller expression difference when compared to nonGCA TABs. Nonetheless, despite a small number of nonGCA subjects, we were able to confirm dysregulation of several miRs in GCA TABs that were identified in other studies with a larger number of nonGCA patients.

In summary, we unraveled new deregulated miR and mRNA molecules in temporal arteries of treatment-naïve patients with GCA and linked their deregulation to temporal artery lumen occlusion and vision disturbances, thereby expanding the knowledge on arterial pathology in GCA.

## 4. Materials and Methods

### 4.1. Study Design, Study Subjects and Clinical Data Collection

The current study included 45 consecutive patients who underwent a complete clinical and laboratory examination, arterial ultrasound imaging and TAB for suspected GCA at the Department of Rheumatology, University Medical Centre Ljubljana, Slovenia from January 2016 to December 2017. GCA diagnosis was established based on the 1990 clinical and laboratory classification criteria of the American College of Rheumatology [70] in combination with a histologically positive TAB or diagnostic halo sign on a temporal artery colour Doppler ultrasonography scan and was confirmed in 41 out of 45 patients. Glucocorticoid therapy commenced after GCA diagnosis, on the same day as TAB was performed. Clinically, patients were examined for the presence of constitutional symptoms (fever, weight loss, fatigue), new onset headache, jaw claudication, visual disturbances, vision loss and signs of the large vessel involvement. The study was conducted in accordance with the Declaration of Helsinki and was approved by the National Medical Ethics Committee of the Republic of Slovenia (approvals #160/07/13, #99/04/15, #65/01/17). All subjects signed the informed consent for participation in the study.

### 4.2. Laboratory Parameters

Among the laboratory parameters, we measured different markers of systemic inflammation, including leukocyte, thrombocyte counts and levels of hemoglobin in peripheral blood (Advia 120, Siemens, Munich, Germany), ESR (WesternGreen method, 1 h), serum levels of CRP, PCT (ADVIA 1800 CRP and PCT assay), fibrinogen (Siemens BCS XP with reagent Multifibren U), ferritin (ADVIA Centaur by direct chemiluminometric technology), haptoglobin, and SAA (immunonephelometry; BN Prospec System, Siemens, Munich, Germany).

### 4.3. Temporal Artery Biopsy

All patients were treatment-naïve prior to TAB and tissue collection. TAB was performed under local anesthesia. The fresh biopsy material was divided into two parts, allowing for histological analyses and gene expression measurements. Tissues used for gene expression analyses were snap frozen in liquid nitrogen until RNA isolation. Histological analyses were performed on formalin-fixed paraffin embedded sections stained with H&E and evaluated by an experienced pathologist. Arterial wall inflammatory infiltrate and arterial occlusion were scored semiquantitatively. Arterial occlusion was considered when luminal stenosis was more than 75%.

### 4.4. Arterial Ultrasound Imaging

Arterial ultrasound included bilateral examination of the facial, vertebral, occipital and temporal (common superficial artery and parietal and frontal branches) arteries, common carotid arteries, axillary arteries and subclavian arteries using the Philips US machine. Using ultrasound, we could accurately visualize only ascendent and abdominal aorta. Because of these limitations, we omitted aortic ultrasound examination from analyses. The imaging was conducted in accordance with the Outcome Measures in Rheumatology (OMERACT) definitions [71]. Briefly, arteries were examined in longitudinal and transversal views with B-mode and colour Doppler examinations. Diagnosis of arteritis was based on detection of a hypoechoic, increased intima-media thickness (a halo sign) and a positive compression sign in temporal and facial arteries and of homogeneous intima-media complex increased thickness in aortic arch arteries. Arterial wall (intima-media) thickness was measured in the common superficial temporal artery in the segment parallel to the skin surface, one cm distal from the point where the artery emerges from deeper tissue levels. Measurements were performed in milimeters on the wall distal to the probe at the defined anatomical area on longitudinal images in systole. Intima-media thickness was measured from the luminal-intimal interface to the medial-adventitial interfaces.

### 4.5. RNA Isolation

3–6 mg of TAB samples (freshly stored in liquid nitrogen) were homogenized using TissueLyser LT (Qiagen, Hilden, Germany) and stainless-steel beads (5 mm) at 50 Hz for 5 min. Total RNA was isolated from TAB samples with RNeasy plus universal mini kit (Qiagen), according to the manufacturer’s instructions, and stored at −80 °C. The concentration and purity of isolated RNA were assessed spectrophotometrically with NanoDropTM 1000 (Thermo Fisher Scientific, Waltham, MA, USA).

### 4.6. MiR Expression Analysis

Reverse transcription was performed with TaqMan™ MicroRNA Reverse Transcription Kit (Thermo Fisher Scientific) at the following conditions: 16 °C—30 min, 42 °C—30 min and 85 °C—5 min. Each 10 µL reaction volume contained 1 µL 10× Reverse Transcription Buffer, 0.67 µL Multiscribe Reverse Transriptase (50 U/µL), 0.13 µL RNAse inhibitor (20 U/µL), 0.1 µL dNTPs (100 Mm), 0.75 µL specific primers for 4 miRNA of interest, 5 µL (20 ng/µL) total RNA template and RNAse-free water. miR expression was measured in duplicates with quantitative real-time PCR (qPCR) analysis, using MicroAmp Optical 96-well reaction plates (Thermo Fisher) and Applied Biosystems 7500 Real time PCR System (Thermo Fisher). Each 10 µL reaction volume contained 5 µL 2× TaqMan Universal PCR Master Mix (Thermo Fisher Scientific), 0.5 µL TaqMan microRNA specific primer, 3.5 µL RNAse-free water and 1 µL (10 ng) cDNA template. PCR cycling conditions were: 95 °C—10 min, followed by 40 cycles at 95 °C for 15 s and 60 °C for 1 min. Data acquisition was performed at the end of each annealing/extension step. MiR primer assays are listed in Supplementary Table S4. Expression of the small nucleolar RNA (RNU48) was used as endogenous control to normalize the data. Data were analyzed with the comparative Ct method and presented as −ΔCt between the Ct of miR of interest and the Ct of endogenous control.

### 4.7. MiR-Target Prediction and Selection of Target Genes

The selection of genes measured was based on identification of target genes of deregulated miRs in GCA patients using computational approach with miRror Suite 2.0. application. MiRror integrates predictions from a dozen of miR resources that are based on complementary algorithms into a unified statistical framework. The resources used include TargetScan, MicroCosm implemented in miRBase, PicTar, DIANA-MicroT, PITA, ElMMO-MirZ, miRanda-based microRNA.org, TargetRank, miRDB and TarBase. MiRror combines the resources following a conversion of the miRs and gene targets identifiers [32]. Our p-value threshold was set to 0.05 and the minimal number of miR–target databases was set to three. MiR-target genes were also selected based on literature search, focusing on regulation of VSMC function.

### 4.8. MRNA Expression Analysis

The expression of mRNAs was measured in 35 histologically positive GCA TABs, 5 histologically negative GCA TABs and four nonGCA control TABs. One negative GCA TAB was omitted from this analysis given the small amount of isolated RNA, sufficient only for miR expression analysis. Reverse transcription of total RNA into cDNA was performed in 20 µL reaction volumes, containing 2 µL GeneAmp 10x PCR Buffer II, 2.2 µL MgCl2 (50 Mm), 4 µL dNTPs (10 Mm), 1 µL random hexamers, 0.5 µL Multiscribe Reverse Transriptase (50 U/µL), 0.4 µL RNAse inhibitor (20 U/µL) (all Thermo Fisher Scientific), 10 µL (40 ng/µL) total RNA template and RNAse-free water. cDNA synthesis was performed at 25 °C for 10 min followed by 30 min incubation at 48 °C and 5 min incubation at 95 °C to heat inactivate the reverse transcriptase. qPCR analysis was performed in duplicates on Applied Biosystems 7500 Real time PCR System (Thermo Fisher Scientific) in MicroAmp Optical 96-well reaction plates (Thermo Fisher Scientific) using self-designed primers (Mycrosynth, Balgach, Switzerland, Integrated DNA Technologies (IDT), Coralville, IA, USA, Supplementary Table S5). Each 10 µL reaction volume contained 6.25 µL 2× FastStart Universal SYBR Green MM (Roche, Basel, Switzerland), 0.375 µL of 10 nM forward and reverse specific primers, 4.5 µL RNAse-free water and 1 µL (10 ng) cDNA template. PCR cycling conditions were: 50 °C for 2 min and 95 °C for 10 min, followed by 40 cycles at 95 °C for 15 s and 60 °C for 1 min. Data acquisition was performed at the end of each annealing/extension step. Expression of *GAPDH* was used as endogenous control to normalize the data. Data were analysed with the comparative Ct method and presented as negative deltaCt between the Ct of gene of interest and the Ct of endogenous control.

### 4.9. STRING Protein-Protein Network Analysis

The STRING Protein-Protein Interaction Networks Functional Enrichment Analysis v11.0 [72] was used to analyse interactions between proteins of genes that were found to be deregulated between TAB positive GCA patients and nonGCA controls. The settings used were full network, where edges indicate both physical and functional protein associations) and medium confidence (0.400) for minimum required interaction score.

### 4.10. Statistical Analysis

Statistical analysis was performed using SPSS statistical software package version 22.0 (SPSS Inc., Chicago, IL, USA) and Graph Pad Prism software 9.0 (Graphpad Software Inc., San Diego, CA, USA). The normality of data distribution was investigated by the Shapiro-Wilk test. Summary statistics are expressed as mean and standard deviation or medians and 25th–75th percentiles (Q_25_–Q_75_). Statistical differences between two groups were calculated using Mann-Whitney U-test or unpaired *t*-test with or without Welch’s correction for continuous variables depending on the normality of data distribution. Multiple group comparisons (TAB positive GCA, TAB negative GCA, nonGCA) were performed by analysis of variance (normal distribution) or Kruskal–Wallis (distribution not normal) test with adjustments for multiple comparisons using Dunn’s post hoc test. Fisher’s exact test was used to test the contingency between categorical variables. For correlation analysis, Spearman’s rank correlation coefficient was calculated. All tests were two-tailed and *p* values of <0.05 were regarded as statistically significant.

## Figures and Tables

**Table 1:** Clinical and laboratory characteristics of GCA patients.

Characteristics	GCA (*n* = 41)
**Clinical symptoms/signs, *n* (%)**	
Symptom duration (days), median (Q_25_–Q_75_)	30 (21–60)
Constitutive symptoms/signs	33/41 (81)
Fatigue	21/41 (51)
Fever	16/41 (39)
Weight loss	24/41 (59)
Sweating	13/41 (32)
Myalgia	5/41 (12)
Arthralgia	1/41 (2)
New onset headache	34/41 (83)
Jaw claudication	26/41 (63)
Visual disturbances	11/41 (27)
Permanent vision loss	7/41 (17)
Polymyalgia rheumatica	6/41 (15)
GCA relapse	10/41 (24)
**Temporal artery biopsy, *n* (%)**	
Transmural inflammation	35/41 (85)
Lumen occlusion	21/41 (51)
**Arterial ultrasound, *n* (%)**	
Temporal artery—halo sign	39/41 (95)
Temporal artery wall thickness (cm), median (Q_25_–Q_75_)	0.069 (0.055–0.090)
Occipital artery	19/41 (46)
Vertebral artery	4/41 (10)
Extended cranial arteritis (≥2 arteries)	28/41 (68)
Large vessel vasculitis	8/41 (20)
Carotid artery	6/41 (15)
Subclavian artery	6/41 (15)
Axillary artery	5/41 (12)
**Systemic inflammation markers, median (Q_25_–Q_75_)**	
ESR (mm/h)	83 (69–108)
CRP (g/L)	102 (48–150)
SAA (mg/L)	294 (124–699)
Ferritin (g/L)	281 (196–525)
Fibrinogen (g/L)	6.4 (6.0–7.4)
Haptoglobin (g/L)	4.8 (3.6–5.9)
Procalcitonin (g/L)	0.07 (0.03–0.10)
Leukocytes (×10^9^/L)	9.2 (7.8–11.5)
Thrombocytes (×10^9^/L)	378 (325–468)
Haemoglobin (g/L)	120 (105–129)

Legend: CRP, C-reactive protein; ESR, erythrocyte sedimentation rate; GCA, giant cell arteritis; SAA, serum amyloid A.

**Table 2:** Analysis of differential frequencies of clinical parameters and ultrasound-based arterial features in patients with GCA.

	Headache	Jaw Claudication	Vision Disturbances	Vision Loss
Extended cranial arteritis	n.s.	0.015	n.s.	n.s.
Vertebral artery	n.s.	n.s.	n.s.	n.s.
Facial artery	n.s.	0.060	0.038	n.s.
Occipital artery	n.s.	n.s.	n.s.	n.s.
Large vessel vasculitis	n.s.	n.s.	n.s.	n.s.
Carotid artery	0.051	n.s.	0.035	0.051
Subclavian artery	0.051	n.s.	n.s.	n.s.
Axillary artery	0.028	n.s.	n.s.	n.s.

Statistical analysis was performed using two-tailed Fisher’s Exact Test with *p*-value of <0.05 considered as statistically significant. Legend: n.s., non significant.

## Data Availability

All relevant data can be found in Supplementary Materials.

## Supplementary Materials

Supplementary materials can be found at https://www.mdpi.com/article/10.3390/ijms22126520/s1.

## Abbreviations

GCA	Giant cell arteritis
GC	Glucocorticoids
TAB	Temporal artery biopsy
TA	Temporal artery
miR	MicroRNA
qPCR	Quantitative polymerase chain reaction
PELI1	Pellino E3 ubiquitin protein ligase 1
YAP1	Yes-associated protein 1
KLF4	Kruppel-like factor 4
MYOCD	Myocardin
PDCD4	Programmed cell death protein 4
SMURF2	SMAD specific E3 ubiquitin protein ligase 2
MMP	Matrix metalloproteinase
TIMP	Tissue inhibitor of matrix metalloproteinase
FGF2	Fibroblast growth factor 2
VEGFA	Vascular endothelial growth factor A
CDS	Colour Doppler ultrasound examination
ESR	Erythrocyte sedimentation rate
CRP	C-reactive protein
SAA	Serum amyloid A
VSMC	Vascular smooth muscle cell
Ct	Threshold cycle
