# Supplementary material for: Dysregulated Expression of Arterial MicroRNAs and Their Target Gene Networks in Temporal Arteries of Treatment-Naïve Patients with Giant Cell Arteritis
Source: Int J Mol Sci. 2021 Jun 17;22(12):6520. doi: 10.3390/ijms22126520 (PMC8234166; doi:10.3390/ijms22126520)
Supplement: Supplementary file 1 [file ijms-22-06520-s001.zip › ijms-1222347-supplementary.pdf]

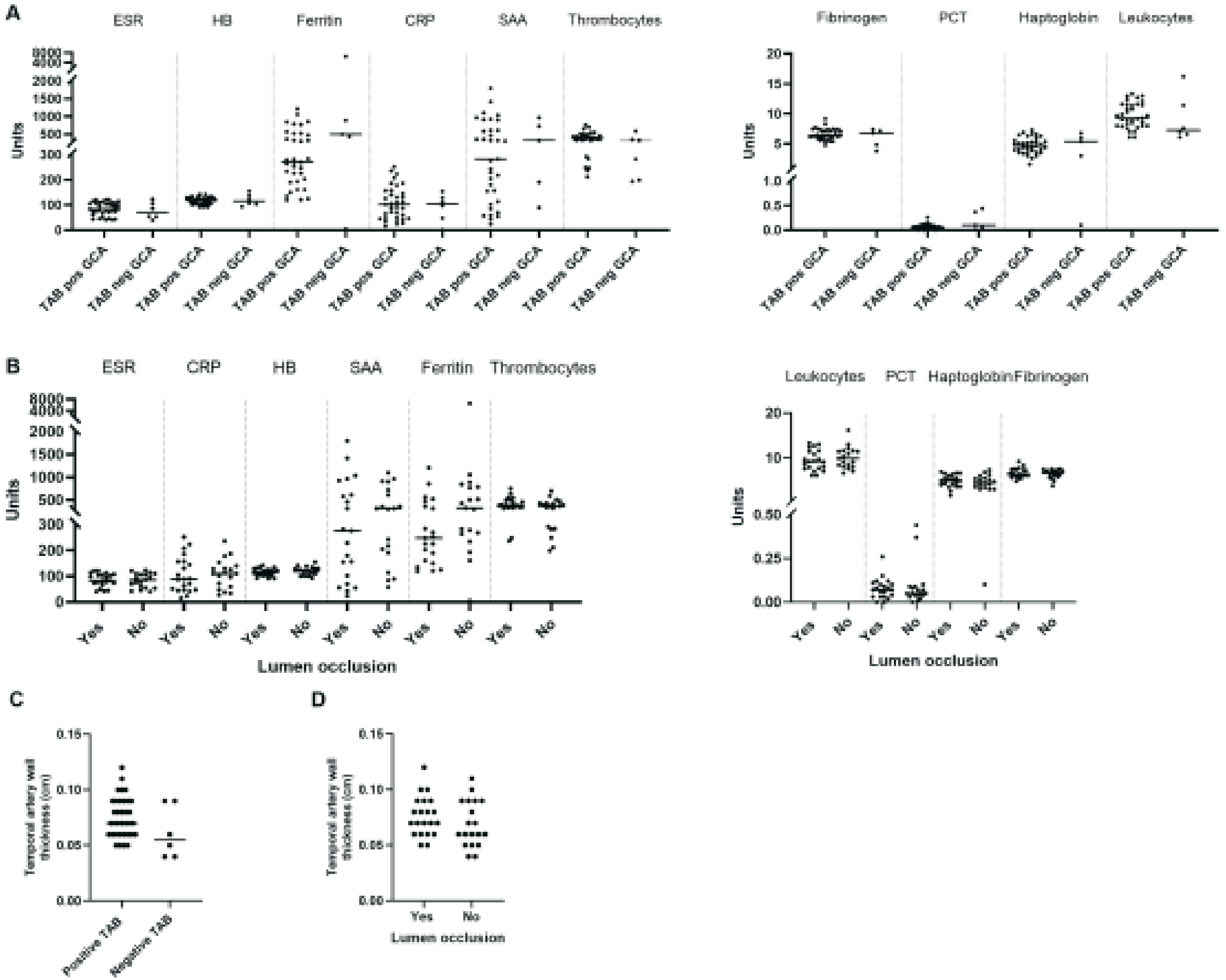
A
ESR
HB
Ferritin
CRP
SAA
Thrombocytes
Units
TAB pos GCA
TAB neg GCA
Fibrinogen
PCT
Haptoglobin
Leukocytes
B
Lumen occlusion
Yes
No
C
Temporal artery wall thickness (cm)
Positive TAB
Negative TAB
D

**Supplementary Figure S1.** Associations of TAB histology with systemic inflammatory markers and sonographic measurements of temporal artery thickness. Lumen occlusion was defined by histological examination of TABs and temporal artery wall thickness was measured by ultrasound. Legend: FGF2, fibroblast growth factor 2; GCA, giant cell arteritis; KLF4, Kruppel-like factor 4; miR, microRNA; MMP, matrix metalloproteinase; PELI1, Pellino E3 ubiquitin protein ligase 1; TAB, temporal artery biopsy; TIMP, tissue inhibitor of matrix metalloproteinase; VEGFA, vascular endothelial growth factor A; VSMC, vascular smooth muscle cells; YAP1, Yes-associated protein 1.

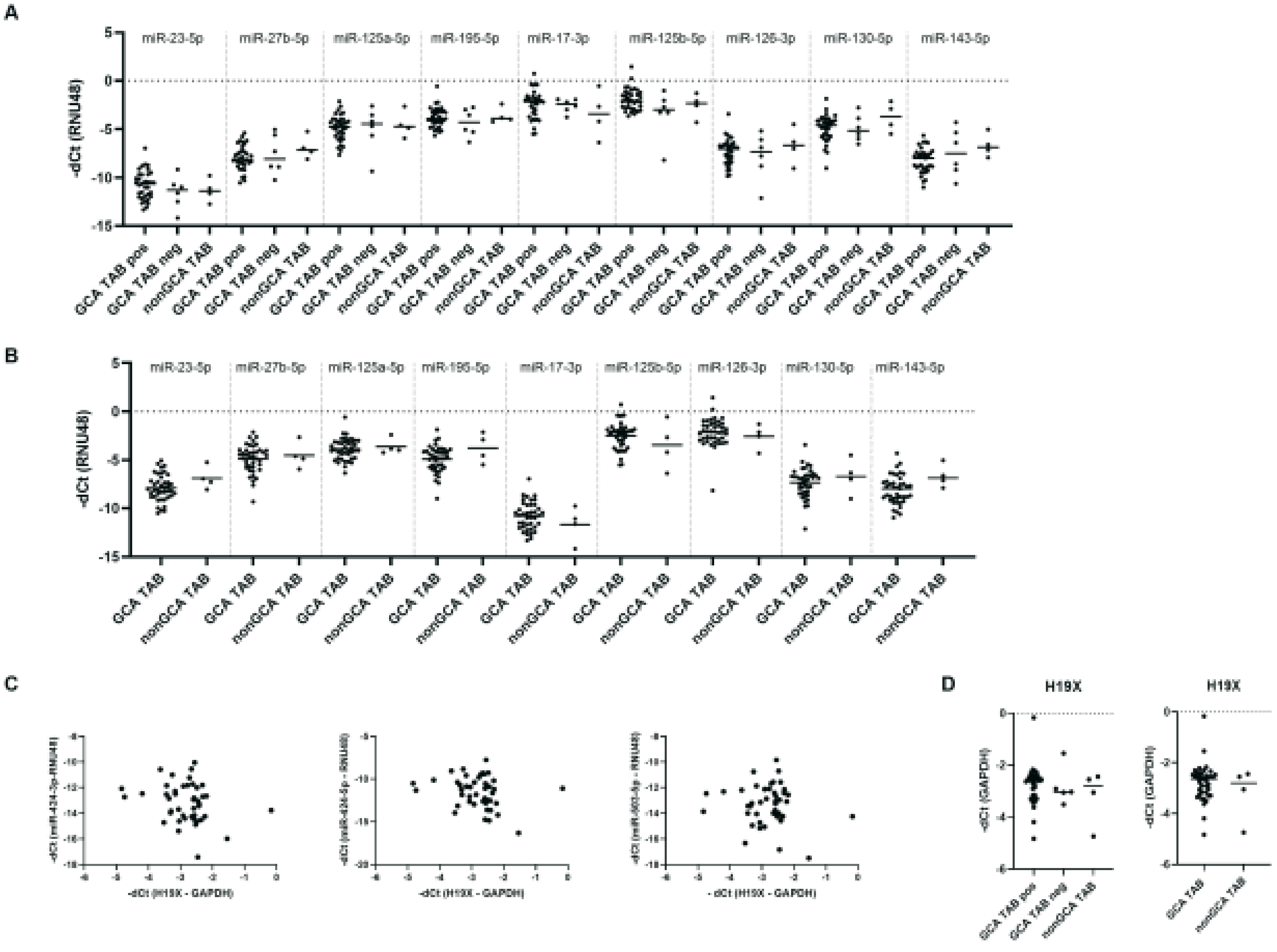


**Supplementary Figure S2.** MicroRNA and *H19X* expression in TABs from GCA and nonGCA patients. Supplementary.

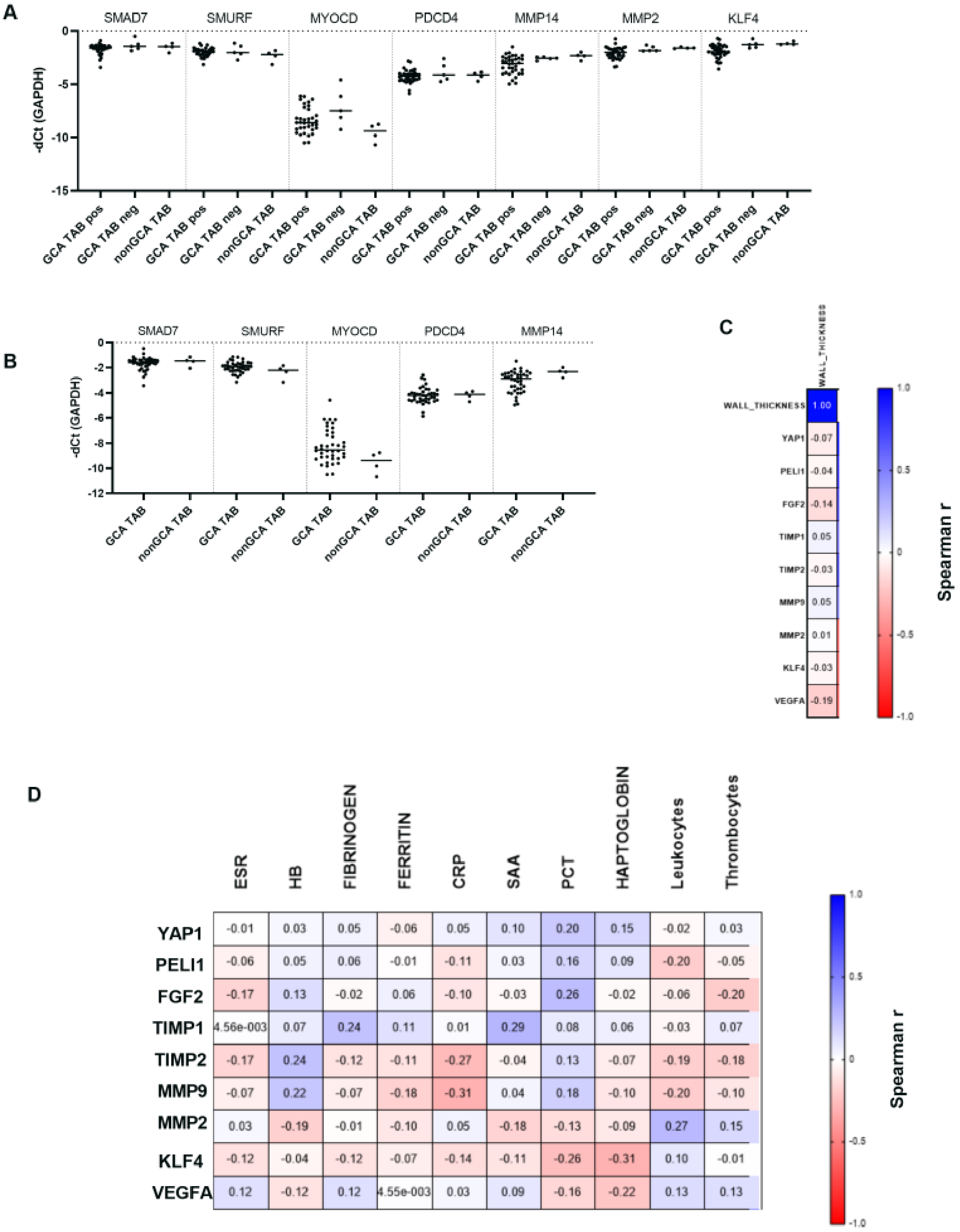


**Supplementary Figure S3.** Expression of miR-target genes and matrix-remodelling genes in GCA and nonGCA patients and Spearman's correlative analysis of their expression with sonographic measurement of temporal artery thickness and systemic inflammatory markers. (A) MiR-target genes which were not differentially expressed between GCA and nonGCA TABs. (B) Matrix-remodelling genes which were not differentially expressed between GCA and nonGCA TABs. (C) Matrix of Spearman's rank correlation coefficients between normalized mRNA expression and sonographic measurements of temporal arterial wall thickness in forty GCA patients. Temporal artery wall thickness was measured by ultrasound. Plotted are miR-target genes and matrix-remodelling genes that were identified as deregulated in GCA TABs. (D) Matrix of Spearman's rank correlation coefficients between normalized mRNA expression and systemic inflammation markers in 35 GCA

patients with histologically positive TABs. Plotted are miR-target genes and matrix-remodelling genes that were identified as deregulated in GCA TABs.
